# To adopt, to adapt, or to contextualise? The big question in clinical practice guideline development

**DOI:** 10.1186/s13104-016-2244-7

**Published:** 2016-09-13

**Authors:** Janine Margarita Dizon, Shingai Machingaidze, Karen Grimmer

**Affiliations:** 1 Faculty of Medicine and Health Sciences, Centre for Evidence-Based Health Care (CEBHC), Stellenbosch University, Francie van Zijl Drive, Tygerberg Cape Town, 7505 South Africa; 2 Center for Health Research and Movement Science, College of Rehabilitation Sciences, University of Santo Tomas, 1018 Manila, Philippines; 3 Cochrane South Africa, South African Medical Research Council, Francie van Zijl Drive, Parow Valley, Cape Town, 7505 South Africa; 4 European and Developing Countries Clinical Trial Partnership (EDCTP), Francie van Zijl Drive, Parow Valley, Cape Town, 7505 South Africa; 5 International Centre for Allied Health Evidence (iCAHE), City East Campus, University of South Australia, P4-18 North Terrace, Adelaide, 5000 Australia

**Keywords:** Clinical practice guidelines, Guideline development, Guideline adoption, Guideline adaptation, Guideline contextualisation

## Abstract

**Aim:**

Developing new clinical practice guidelines (CPGs) can be time-consuming and expensive. A more efficient approach could be to adopt, adapt or contextualise recommendations from existing good quality CPGs so that the resultant guidance is tailored to the local context.

**Results:**

The first steps are to search for international CPGs that have a similar purpose, end-users and patients to your situation. The second step is to critically appraise the methodological quality of the CPGs to ensure that your guidance is based on credible evidence. Then the decisions begin. Can you simply **‘adopt’** this (parent) clinical practice guidelines, and implement the recommendations in their entirety, without any changes, in your setting? If so, then no further work is required. However this situation is rare. What is more likely, is that even if recommendations from the parent clinical practice guidelines can be adopted, how they are implemented needs to address local issues. Thus you may need to ‘**contextualise’** the guidance, by addressing implementation issues such as local workforce, training, health systems, equipment and/or access to services. Generally this means that additional information is required (Practice/Context Points) to support effective implementation of the clinical practice guidelines recommendations. In some cases, you may need to **‘adapt’** the guidance, where you will make changes to the recommendations so that care is relevant to your local environments. This may involve additional work to search for local research, or obtain local consensus, regarding how best to adapt recommendations. For example, adaptation might reflect substituting one drug for another (drugs have similar effects, but the alternative drug to the recommended one may be cheaper, more easily obtained or more culturally acceptable). There is lack of standardisation of clinical practice guidelines terminology, leading clinical practice guideline activities often being poorly conceptualised or reported. We provide an approach that would help improve efficiency and standardisation of clinical practice guidelines activities.

## Background

The volume of literature available to support the construction of new (de novo) evidence-based clinical practice guidelines (CPGs) was recently highlighted by Schünemann et al. in a recent comprehensive international review of the content of 35 guideline development manuals [1]. This volume of literature is not matched by research into updating [2, 3] or adapting/contextualising CPGs [4–7]. Thus it would seem that de novo CPG development is the preferred approach, when clinicians, managers or policy makers are faced with clinical issues of local importance.

However international CPG repositories are generally freely available via the internet [8–11] and a simple search highlights a wealth of good quality CPGs already written, for a large number of clinical conditions. One could query then, why clinicians, managers or policy makers might develop yet another local CPG, when so many already exist? One answer may be that if a CPG group decides to use guidance developed by others, there are usually immediate challenges related to putting it into practice, largely reflected by the question of whether the CPG can be effectively implemented in a new setting [12–14]. There are many issues which influence effective CPG implementation, including but not limited to comprehensiveness and currency of the evidence-base, acceptance by local policy-makers, clinicians and/or patients, cultural relevance, local contexts, availability of care, affordability, equity and access [15]. Thus it is perhaps understandable why CPG groups in particular settings choose to develop de novo CPGs, rather than use CPGs already written by others.

De novo CPG development (‘from scratch’) is usually an expensive and time-consuming business, requiring dedicated teams of methodologists and experts (e.g. clinicians, managers, policy-makers, consumers), who search, critique and debate the usefulness and relevance of the body of evidence which could provide relevant clinical guidance. Consequently, the financial, human resource and opportunity costs of de novo CPG development are often outside budgets of low-to-middle income countries (LMICs). Moreover, the disease burden in these countries is often higher than in middle-to-high income countries, and thus a focus on evidence-based disease management is often even more urgent, to minimise wastage and ensure optimal care for optimal cost [16]. Thus in LMIC countries, building on CPGs which have been developed elsewhere, and using a structured process to make recommendations relevant to local contexts might not only be a persuasive alternative to undertaking de novo CPG activities, but also a way of breaking down barriers to implementation.

This paper proposes ways to consider the need for de novo CPG development when already available good quality CPGs might be sourced from elsewhere, and modified to provide guidance appropriate for local contexts.

## Our approach

### Critical elements of clinical guidance

In a paper we recently published, we proposed a CPG classification system [the ‘South African Guidline Evaluation (SAGE) Clinical Practice Guideline Development Framework’] that has a base of transparent evidence synthesis processes (tier one); layered with clinical contexts (tier two); which in turn supports end-products tailored specifically for different contexts, users and purposes (tier three)—classified as ‘evidence-based summary recommendations’, ‘patient management tools’, or ‘protocols’.

#### Tier 1 (evidence base)

The evidence base forms the foundation of all forms of clinical guidance. Without this tier, there is little to support the credibility of recommendations in terms of ‘what the evidence says’. Producing the evidence base is usually the domain of methodologists, who establish what literature is available to answer clinical questions in the CPG, and how believable the evidence is. The GRADE group produced a widely-used set of instructions to classify the strength of the underlying evidence for CPG recommendations [very strong (benefit/risk trade-off unequivocal, high quality evidence, 1A) to the very weak (benefit/risk questionable, low quality evidence, 2C)] [17, 18]. To be credible, the evidence base should be derived from transparent, comprehensive literature reviews relevant to guideline questions, following the steps outlined by Schünemann et al. [1].

#### Tier 2 (expert input)

Evidence derived from experts is not always dependable. While expert opinion is an essential element in the Evidence-based Medicine model proposed by Sackett et al. [19], it needs to be in addition to the body of evidence, not instead of it. Experts may well present a comprehensive understanding of the available research base, however they may also provide opinion that is without the backing of independent evidence reviews. Expert opinion alone runs the risk of presenting selective, non-current or misleading (biased) views of the available evidence [20–22]. However, in circumstances where there are evidence gaps (no research has been conducted, or the research is of questionable value), expert opinion is recognised as a credible evidence source (SIGN guideline developers handbook) [23]. Expert opinion garnered using robust qualitative research such as Delphi studies, provides credible ‘best available evidence’ statements in the absence of sound research evidence [22].

The second CPG tier we proposed requires expert input, as this layer is essential in determining relevance and applicability of research evidence to local contexts. Local contexts deal with a range of issues that are often not addressed or reported in the body of research, such as local systems, and operational issues such as funding and funding priorities, historical health service delivery, health priorities, health workforce type, training, mobility and availability, how decisions are made, available human and infrastructure resources, burden of disease, and patient need. In a CPG team, determining the second layer of CPGs is usually the domain of lead clinicians, managers, policy-makers, funders and end-users (usually patients and local clinicians). However, there is little in the CPG literature regarding how to comprehensively address the relevance and applicability of evidence, to local contexts. FORM is one of the few tools that provides guidance when considering the contexts of recommendations, regarding applicability to end-users and patients, and relevance to local healthcare environments [24]. This second tier usually results in ‘Context Points’ which enrich the Tier 1 findings, and provide information to assist local users to apply the research evidence.

#### Tier 3 (end-user guidance documents)

This is the least well explored in the literature, and it refers to the way that guidance is presented to address end-user needs (i.e. in short form evidence summaries, patient management tools, decision-making algorithms, or protocols to do specific tasks). How recommendations are presented has a significant impact on evidence-uptake and compliance [13, 22].

Considering the cost, time and human resource implications of de novo CPG development, we suggest that CPG groups should consider alternatives to de novo development activities. To assist CPG groups particularly in LMICs to be effective and efficient, we propose an approach to **Adopt, Contextualise** or **Adapt CPGs**, using an existing high quality evidence base from international guidelines developed in other countries for the same target patient population and the same end-users. CPG groups should rather not focus on recreating Tier 1 in the CPG development framework [25], but instead focus on Tier 2, where they can harness local experts with local knowledge to complement the existing evidence-base, and produce Tier 3 outputs that provide locally relevant and ready to implement recommendations.

### To adopt, contextualise or adapt?

**Fig. 1:**
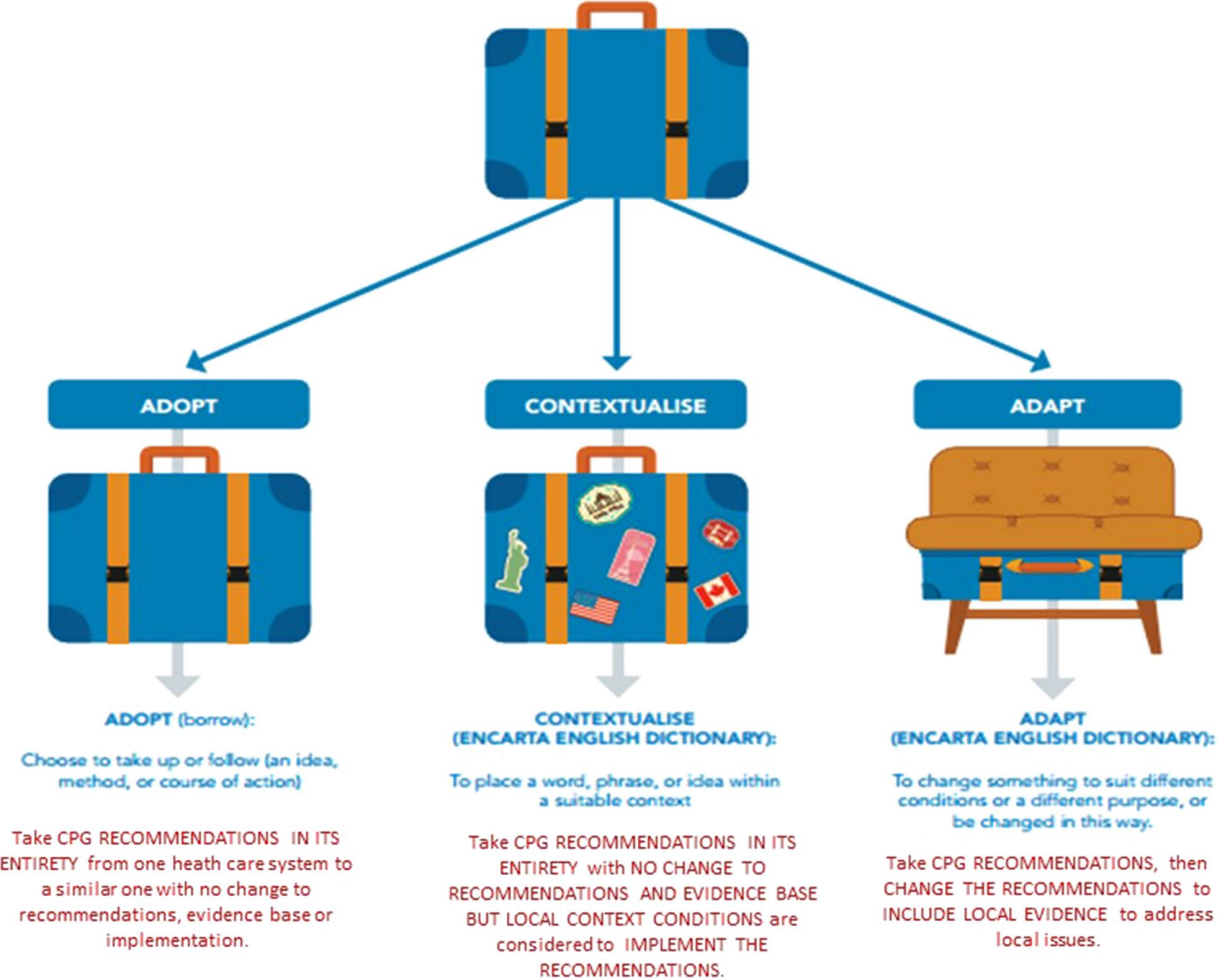
Suitcase analogy for adopt, adapt and contextualise

**Adopting** refers to something being accepted and put into use without any change [e.g. a suitcase borrowed and being used as it is (Fig. 1)] and with (usually) the intention of returning it in the same condition. Many diseases/health problems occur commonly around the world (e.g. stroke, cancer, asthma, diabetes, hypertension, back pain). There may be country-to-country variations in prevalence, however it is reasonable to expect that research into these conditions would be conducted, and published, by researchers around the world. It is also reasonable that comprehensive search strategies developed for Tier 1 [25] of a de novo CPG would identify all relevant international literature, and not just that from the country where the CPG is being developed. Thus, for a given condition, a good quality CPG developed in UK, for instance, could include the same literature as a good quality CPG developed at the same time in Australia, even though the two CPG development groups may not be aware of each other’s activities. One could argue therefore, that where a current, good quality CPG with a rigorous, defensible evidence base (Tier 1) is already available, it is unnecessary and inefficient to redo the whole development process simply to be seen to have a locally-developed CPG.

Following this line of thought, adopting a CPG developed elsewhere means a commitment to implementing its recommendations exactly as proposed, without change or caveat, in a new setting. Thus all three tiers of a CPG developed in Australia for instance, for the management of a particular condition, may be adopted by another similar country with a similar healthcare system, similar patient types, and similar economy, with the expectation that the recommendations will be equally as applicable, relevant and effective as they were in the parent country, in underpinning good processes and producing desired outcomes.

**Contextualising** a CPG occurs when a CPG produced elsewhere is also adopted in its entirety, but to implement it effectively requires caveats and/or additional considerations, to address local contexts. Contextualisation generally relates to local service delivery issues. Considering the suitcase analogy (Fig. 1), contextualisation occurs when a suitcase is borrowed, but then contextualised, by the addition of travel stickers, or additional locks, or wheels. Whilst it is still a suitcase, it is not the same as the original suitcase. Thus during contextualisation, the evidence base (Tier 1), and the resultant recommendations (Tier 3) remain the same as the original ‘borrowed’ guideline (i.e. they are adopted), however additional Tier 2 processes (expert input) are essential in order for the recommendations in Tier 3 to be effectively implemented locally. The CPG contextualisation activities undertaken by Philippines Academy of Rehabilitation Medicine (PARM) are a case in point [6]. The PARM group found that there was no need to recreate existing guidance for the management of stroke, however effective implementation of international CPG recommendations in its current setting required consideration of local contexts of workforce type, availability and training, patients’ physical access to care, local resources, referral systems, workforce hierarchy and record keeping. An example of this occurred when the PARM group adopted the recommendation for the use of the water swallow test for diagnosing dysphasia in acute stroke, a recommendation from the SIGN (2010) guidelines [26]. PARM’s contextualisation approach was to layer this recommendation with specific context points in order to ensure effective Filipino implementation [27]. The PARM context points addressed diagnostic tools and requisite equipment, workforce available and training required to administer the test, and specifications of when the water swallow test should be conducted [28]. These were mapped against minimum standards of care possible (in most settings) and additional standards of care (in advanced settings) enabling the provision of best practice nation-wide [*PARM STROKE GUIDELINES* (http://www.eparm.org/images/STROKE-Guideline.pdf, page 118] [28] (see Table 1).

**Table 1:** Dysphagia assessment (reproduced from PARM Stroke Guidelines with permission

Context considerations	Minimum standard care of practice	Additional standard care of practice
Diagnostic tools	Water swallow test Standardized clinical bedside assessment Pulse oximetry	Videofluroscopy-modified barium swallow test (VMBS) and/or fiberoptic endoscopic evaluation of swallowing (FEES)
Equipment	Water, food of different consistencies (pudding and buscuits-deleted), spoon, cup, stethoscope (see Appendix 11) Pulse oximeter	Videofluroscopy machine Fiberoptic endoscopy machine
Workforce	Physiatrist Occupational therapist Nurse	Radiologist Otorlaryngologist Speech pathologist
Resources	Protocol for water swallow test (Appendices 8 and 9) Protocol for standardized clinical bedside assessment (Appendix 10)	Protocol for barium swallow and FEES when it is considered to be pathological
Training	Training needed for water swallow and standardized clinical bedside assessment	Specialist training in tertiary hospital
When is it done	As screening tool for aspiration Before nasogastric tube is removed or anything to be given by mouth	Done after a failed water swallow test, or presence of signs and symptoms of aspiration

Context points of minimum and additional standard care of practice for dysphagia, Table 64) [28])

Used with permission from PARM

**Adapting** a CPG refers to changing the CPG recommendations to address local issues. This is a complex issue, where Tier 1 in the CPG may (or may not) be changed (depending on whether the guidelines questions remain relevant in the new setting), and adaptation may occur within the Tier2 process, to revise the way recommendations are worded or presented in Tier 3. Not adapting may mean that the CPG recommendations may be rendered useless in a new setting. The analogy of the suitcase ‘chair’ is provided (Fig. 1), where the suitcase no longer retains its original state, and has since been modified. An example might be found in recommendations for a specific drug regimen. Whilst a recommendation from an international, good quality CPG might be to use a particular drug in a high income country for a specific purpose, it may not be possible for this same regimen to be followed in a LMIC because of cost, or availability of the recommended drug, or contra-indications with other drugs commonly administered there. Another drug may be substituted for the recommended drug, because it is cheaper, more easily obtained or more locally acceptable. The substitute drug might also be better able to be stored in LMIC conditions, and may retain its potency or shelf-life better than the initially recommended drug. There may be a trade-off in effectiveness or dose, in order that local health care providers can still deliver the best available local practices, in the face of cost, geographic, supply or cultural constraints.

Adopting, contextualising and adapting CPG recommendations may all be relevant, at the same time, within the one CPG, depending on which recommendations are relevant to the users of the CPG. For instance, recommendations regarding diagnosis (i.e. signs and symptoms) for a condition may be readily adopted, whilst recommendations for management (i.e. interventions such as drugs or highly specialised care management) may require contextualisation and/or adaptation to be actionable locally.

## Discussion

**Fig. 2:**
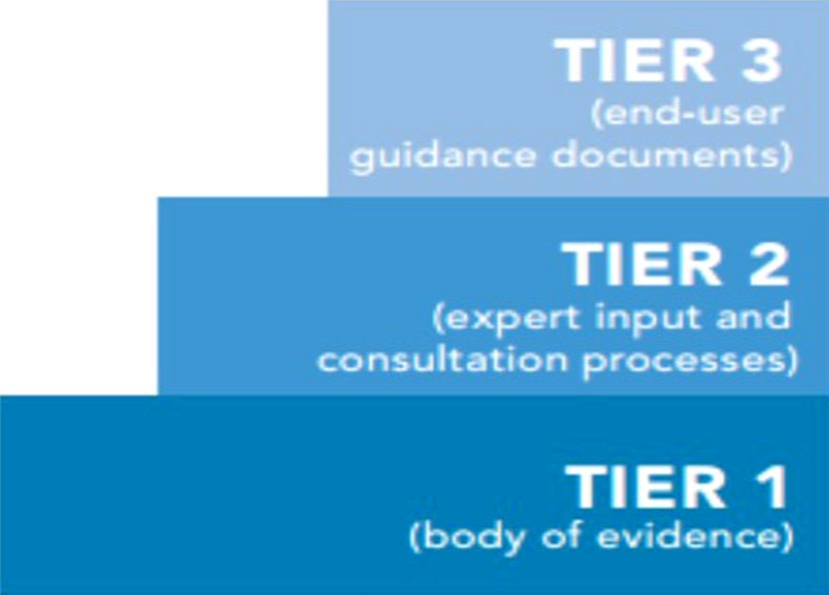
SAGE CPG developmnet framework

Research into CPGs has escalated over the last 15–20 years, with a concomitant increase in theories and methods [1–7]. Terminology has also increased in volume and sophistication, although currently CPG terms can have different meanings [29]. The issue of contextualisation and adaptation is a case in point. The two terms are used interchangeably although they have quite different connotations (as outlined in Fig. 2), and depending on the approach taken, require different CPG activities. We contend that to contextualise is a component of adopting a CPG (by addressing local implementation issues without changing the CPG recommendations), whereas to adapt a CPG requires permanent change, perhaps additional literature searches to identify local information to support substitution, or change of ‘parent’ CPG recommendations, to ensure that CPG recommendations are relevant to local contexts, resources and/or culture.

Best known research into transferring CPGs from one setting to another is the work of the ADAPTE Collaboration, which reported 24 steps in adapting CPG recommendations from one setting to another process [5, 7]. This group defines adaptation as ‘*the systematic approach to considering the use and/or modification of a guideline developed in one cultural or organizational setting for application in a different context. Adaptation can be considered as an alternative to de novo guideline development …….*

*The adaptation process…….has been designed to ensure that the final recommendations address specific health questions relevant to the context of use, and address the needs, priorities, legislation, policies and resources in the target setting without undermining the validity of the target recommendations*’ (Adaptation Resource Kit 2009 p9) [7]. We believe that the ADAPTE group presents an amalgam of adaption, adoption and contextualization in their resource kit. The ADAPTE framework underpinned the innovative Filipino guidelines contextualisation project [6]. PARM recognized that it did not have the time, finances, and expertise or, in fact, need, to develop Filipino-relevant de novo guidelines. Thus a dedicated band of volunteers embarked on the process of using others’ work to inform their practices. This group recognized and addressed the lack of detail in the ADAPTE process on ‘how to’ transfer recommendations from guidelines developed in high income country settings to a LMIC setting, where healthcare policy and contexts, funding, workforce, resources and training were significantly different from those in the parent CPGs. This particularly reflected gaps between Steps 14 and 17 in the ADAPTE resource manual, relating to just how to take recommendations from one setting and put them into place in another (Table 2). The PARM group debated and differentiated between the notions of ‘adaptation’ and ‘contextualisation’, particularly regarding whether changes were to be made to the parent CPG. The PARM group proposed an innovative contextualisation approach of mapping multiple relevant best-practice guideline recommendations into a typical Filipino patient pathway, and then developing local ‘context points’ relevant to Filipino healthcare settings to support seamless uptake and implementation of best evidence [6]. This work has since been recognized as best practice for LMIC by the International Society of Physical and Rehabilitation Medicine (ISPRM), as a practical cost-effective and efficient alternative approach to developing local context de novo CPGs [30].

**Table 2:** ADAPTE vs PARM approach

ADAPTE	PARM contextualisation process	
Step 1 establish an organising committee	Implicit—purpose-driven	
Step 2 establish a guideline topic	Implicit—purpose-driven	
Step 3 check whether adaptation is feasible	Implicit—purpose-driven	
Step 4 identify necessary resources and skills	Step 1 training	
Step 5 complete tasks for set up phase		
Step 6 write adaptation plan	Step 2 establish ‘usual’ patient journeys	
Step 7 scope and purpose (determine the questions)	Step 3 establish scope and purpose	
	Step 4 establish a work plan and working groups	
Step 8 search for guidelines and other relevant documents	Step 5 search for appropriate guidelines	
Step 9 screen retrieved guidelines	Step 6 screen guidelines relevant to patient journeys and identifying relevant ones	
Step 10 reduce a large number of retrieved guidelines		
Step 11 assess guideline quality	Step 7 critically appraise guideline quality and currency and retain relevant high quality guidelines	
	Step 8 contact developers for permission and to undertake external review of completed synthesised guidelines	
Step 12 assess guideline currency		
Step 13 assess guideline content	Step 9 summarise differences between guidelines in wording of recommendations, ways of reporting underpinning evidence, and summarising strength of the evidence	
Step 14 assess guideline consistency		
	Step 10 identify recommendations relevant to steps along the patient journey Step 11 develop a process for dealing with 2 or more relevant recommendations	
	Step 12 develop PARM Writing Guide	
Step 15 assess acceptability and applicability of recommendations	Step 13 write PARM endorsements based on strength of evidence	
Step 16 review assessments		
Step 17 select between guidelines and recommendations to create an adapted guideline	Step 14 consider applicability and generalizability of recommendations to Filipino situations (NHMRC FORM) using PARM context points	
Step 18 Prepare draft adapted guideline	Step 15 map the PARM endorsements and Context Points for collated recommendations into the patient journey	
	Step 16 collate guideline chapters and edit for consistency	
	*See Step 17 congruent with Steps 13*–*15*	Step 17 develop an implementation plan congruently with Steps 13–15
Step 19 external review	Step 18 present guideline at national meeting	
Step 20 consult with endorsement bodies		
Step 21 consult with source guideline developers	Step 19 undertake focused public consultation including seeking suggestions for additional PARM Context Points	
Step 22 acknowledge source documentation		
Step 23 plan for aftercare of adapted guideline		
Step 24 produce final guideline document		
	Step 20 plan and evaluate the guideline roll out	
	Step 21 establish partnerships	

The differences between the ADAPTE framework and the PARM group’s processes are outlined in Table 2.

## Conclusion

CPGs are integral to the delivery of best practice care. The work required to develop and update the evidence base underpinning CPGs needs to be ongoing, to ensure currency of the evidence base underpinning recommendations. However the utility, applicability and relevance of recommendations to local settings requires significant investment from local experts and opinion leaders [25]. Clear decisions about using existing evidence sources, and adopting (with or without contextualising) or adapting, offers persuasive ways forward for CPG groups, to ensure that scarce resources are focused on implementation. CPG terminology will continue to evolve, and gain greater clarity, as more groups become engaged with the processes underlying putting the best evidence into practice in ways which address local need.

## Abbreviations

CPG: clinical practice guideline
LMIC: lower middle income countries
SAGE: South African guideline evaluation
GRADE: grading of recommendations, assessment, development and evaluations
PARM: Philippines Academy of Rehabilitation Medicine
